# Evaluation of the vitamin D and biomedical statuses of young children with β-thalassemia major at a single center in southern China

**DOI:** 10.1186/s12887-019-1744-8

**Published:** 2019-10-23

**Authors:** Uet Yu, Li Chen, Xiaodong Wang, Xiaoling Zhang, Yue Li, Feiqiu Wen, Sixi Liu

**Affiliations:** Department of Hematology and Oncology, Shenzhen Children’s Hospital, Yitian Road No. 7019, Futian, Shenzhen, 518038 Guangdong China

**Keywords:** Thalassemia, Iron overload, Vitamin D deficiency

## Abstract

**Background:**

In young children, β-thalassemia major (β-TM) is associated with potentially severe clinical characteristics, including poor growth, feeding difficulties, hepatosplenomegaly, bone metabolic disorders, and skeletal abnormalities.

**Methods:**

In this study, we reviewed the demographic and clinical characteristics (e.g., age, sex, duration of blood transfusion and chelating therapy, and vitamin supplementation) and serum biomarker levels (e.g., iron accumulation, bone metabolism, liver, kidney, and thyroid function markers) of 32 patients that received regular blood transfusion at a single center in southern China with the aim of stratifying the risk of severe complications such as osteopenia, endocrinopathies, and multi-organ failures.

**Results:**

Although all patients exhibited moderately to strongly elevated serum ferritin levels, this biomarker was significantly higher in children older than ≥5 years, compared to younger children (*p < 0.05, 1512 ± 192.6 vs. 2337 ± 299.8 ng/ml, Mann-Whitney *U* test). Older children had a significantly lower 25-hydroxy vitamin D3 (25(OH)D_3_) level, compared to younger children (**p < 0.01, 34.25 ± 11.06 vs. 23.05 ± 9.95 ng/ml, Mann-Whitney *U* test). No age-related differences were observed in serum calcium, phosphorus, and PTH levels. Regarding liver function, the serum alanine aminotransferase (ALT) level was significantly increased in children older than ≥5 years, compared to younger children (*p < 0.05, 19.17 ± 2.44 vs. 43.45 ± 9.82I U/ml, Mann-Whitney *U* test). However, no age-related differences were observed in the serum levels of other liver or kidney and thyroid biomarkers.

**Conclusions:**

Our results suggest that in older children, hepatic iron overload may be associated with a low serum concentration of 25(OH)D_3_, an indicator of vitamin D deficiency and altered bone metabolism. Iron accumulation may also be associated with a higher concentration of ALT, a sensitive marker of liver malfunction. These findings may provide important clinical indications of the need for intervention to prevent severe complications in children with β thalassemia.

## Background

Thalassemia is a genetic disorder characterized by the complete absence or reduced synthesis of the alpha- or beta-globin chain of hemoglobin. Although thalassemia is usually asymptomatic or associated with only mild anemia, patients with severe disease require lifelong blood transfusions for survival [1]. The term beta-thalassemia, also known as beta-thalassemia major (β-TM), encompasses several of the most common genotypes associated with blood transfusion-dependent thalassemia. Patients with β-TM are homozygous or compound heterozygous carriers of beta^0^ or beta^+^ genes [2]. Most such patients develop symptoms of β-TM during early childhood, generally between the ages of 6 and 24 months. These clinical symptoms may include poor growth, feeding difficulties, hepatosplenomegaly, bone metabolic disorders, and skeletal abnormalities. Children with β-TM must receive regular blood transfusions to prevent severe complications and maintain normal physiological growth [2, 3].

Despite the life-saving nature of long-term blood transfusion, iron toxication due to dysregulated cellular iron metabolism is the leading cause of prolonged complications in patients with β-TM [4, 5]. Normally, iron is stored intracellularly in the form of ferritin. Under conditions of iron overload, excess iron accumulates within tissues such as the liver, heart, lungs, and endocrine glands. These unbound iron particles contribute to the release of free radicals, which damage membrane lipids and other macromolecules and lead to cell death and, eventually, organ failure [6, 7]. In recent decades, modified blood transfusion protocols and chelating therapy have greatly improved the life expectancies and quality of life of patients with β-TM. However, treatment with high doses of iron chelators, such as desferrioxamine (DFO), may exacerbate complications such as osteopenia and osteoporosis [5, 8, 9].

Guangdong Province in southern China has one of the highest incidences of β-TM in the world. Here, Shenzhen Children’s Hospital (SZCH), which is located in the second-largest city (population: 10 million), houses the only pediatric center in the province. In this study, we retrospectively reviewed the data of 32 children who visited SZCH for regular blood transfusions between January and June 2018. After summarizing the clinical and biomedical data of these patients, we investigated age-related differences in these parameters.

## Methods

### Patient recruitment

β-TM patients who were admitted to the Department of Hematology and Oncology at SZCH, Guangdong, China between January and June 2018 were recruited for this study. The study protocol was approved by the Ethics Research Committee at SZCH and conducted according to the ethical standards of the Committee on Publication Ethics (COPE). Written consent was obtained from the parents of the included patients before the study.

The following patient inclusion criteria were applied: 1) a homozygous or double heterozygous β-TM status based on a genetic evaluation, 2) requirement for regular blood transfusion to maintain a hemoglobin level > 90 g/L, 3) diagnosed β-TM and regular follow-ups at SZCH. Patients 1) with other genotypes associated with blood transfusion-dependent thalassemia, 2) who underwent hematopoietic stem cell transplantation during follow-up, 3) whose parents did not provide written consent, and 4) who left the study during the follow-up were excluded from the analysis. The following data were collected from the included patients: demographic characteristics; age at β-TM diagnosis, duration of blood transfusion, use of chelating therapy; duration of chelating therapy; use of vitamin D and calcium supplements; and laboratory examinations of serum biomarkers.

### Laboratory assessment

All laboratory assessments were performed at the medical diagnostic lab at SZCH. The serum ferritin levels were examined in all patients. Laboratory evaluations of bone metabolism included measures of the serum 25-hydroxy vitamin D3 (25(OH)D_3_), phosphorus, calcium, and parathyroid hormone (PTH) concentrations. Liver function was assessed by measuring the serum concentrations of alanine aminotransferase (ALT), aspartate aminotransferase (AST), total bilirubin (TBIL), total protein (TP), and albumin (ALB). Kidney function was examined by measuring the serum concentrations of creatinine (Cr) and blood urea nitrogen (BUN). Thyroid function was assessed by measuring the serum concentrations of triiodothyronine (T3), thyroxine (T4), and thyroid-stimulating hormone (TSH).

### Statistics

The statistical analyses were performed using Prism software (GraphPad, Inc., La Jolla, CA, USA). Unpaired Student’s *t* test was performed. A *p* value < 0.05 was considered statistically significant.

## Results

The demographic characteristics of the recruited patients were retrospectively reviewed (Table 1). The study included 19 boys and 13 girls who continued to participate in follow-up evaluations throughout the study period. The patients ranged in age from < 1 to 12 years old (yo), with a mean age (± standard deviation) of 5 ± 3 yo. Thirteen patients were < 5 yo, while 19 were ≥ 5 yo. Most patients had been diagnosed with β-TM at an age < 2 yo, and the mean age at the time of diagnosis was 14 ± 15 months.

**Table 1:** Demographic characteristics of the patients

	No. of patients
Total No. of patients	32
< 5 years old	13
≥ 5 years old	19
Sex	
Male	19
Female	13
	Mean ± SD
Mean age (years)	5 3
Mean age of diagnosis (months)	14 15
Mean age of first blood transfusion (months)	15 15
Mean age of the start of chelating therapy (years)	3 2
	No. of patients
Chelating regulatory (≥1 chelating agent)	
Regular	19
Only Deferoxamine	0
Only Defriprone	6
Only Deferasirox	6
Deferoxamine+Defriprone	4
Defriprone+Deferasirox	2
Not regular	1
Never	12
No. of patients with oral supplementations (Calcium or Vitamin D)	
Regular	12
Occasional	5
Never	15

Children with confirmed β-thalassemia major who visited the Department of Hematology and Oncology Department at Shenzhen Children’s Hospital for regular blood transfusions between January and June 2018 were recruited for the study. The patients’ demographic characteristics were reviewed retrospectively

All patients received regular blood transfusions at a volume of 15 ml/kg at intervals of 2–4 weeks to maintain a hemoglobin level > 90 g/L, and most had received the first blood transfusion almost concomitantly with the diagnosis. Nineteen of 32 patients received regular iron-chelating therapy with at least one chelation agent, and one patient received occasional iron-chelating therapy. The remaining 12 patients, including five patients younger than 5 yo, had no history of chelating therapy. Only 12 patients reported the regular oral intake of calcium or vitamin D supplements, and five reported occasional calcium or vitamin D supplement use.

**Fig. 1:**
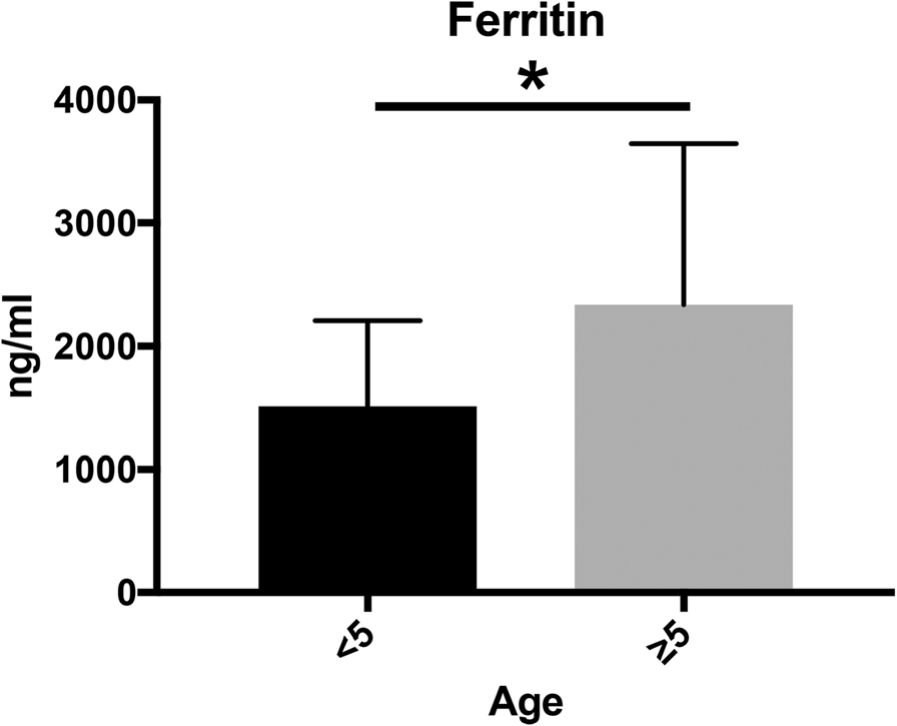
Age-related differences in the serum ferritin concentrations of patients with β-thalassemia major. Patients ≥5 years of age had significantly higher serum ferritin concentrations, compared to patients < 5 years of age (*p < 0.05, 1512 ± 192.6 vs. 2337 ± 299.8 ng/ml, Mann-Whitney *U* test)

All children recruited for this study exhibited moderate to severe iron overload, with a mean serum ferritin level of 2002 ± 1161 ng/ml. Consistent with many previous studies, the severity of iron overload was associated with age [8, 10–12]. Accordingly, we divided the patients into two age groups, < 5 vs. ≥5 yo. A comparison revealed significantly higher serum ferritin concentrations in the older children, compared to the younger children (*p < 0.05, 1512 ± 192.6 vs. 2337 ± 299.8 ng/ml, Mann-Whitney *U* test) (Fig. 1).

Iron accumulation in tissues is associated with functional dysregulation in many organs, including the liver, kidney, and endocrine organs such as the thyroid and pancreas. We did not evaluate pancreatic function in this study because young children find it difficult to comply with oral glucose tolerance testing. However, a random sampling of blood glucose levels revealed values within the normal ranges in both age groups. We additionally evaluated serum biomedical markers of liver function, bone metabolism, kidney function, and thyroid function and compared these values to the normal ranges according to the laboratory standards of the medical diagnostic lab at SZCH. Notably, only the overall mean serum concentration of 25(OH)D_3_, 28.3 ± 10.9 mmol/L, was slightly below the laboratory standard (30 mmol/L) (Table 2).

**Table 2:** Laboratory evaluations of the patients

	Mean	±SD	Normal range
Ferritin (ng/ml)	2002	1161	22–322
Liver function			
ALT (IU/L)	34.3	36.8	0–40
AST (IU/L)	37.3	19.9	0–40
TBIL (μmol/L)	21	10.9	0.9–17.1
TP (g/L)	64.3	4.47	46–80
ALB (g/L)	40.1	2.03	35–55
Bone metabolism			
25(OH)D3 (ng/ml)	28.3	10.9	30–100
Ca (mmol/L)	2.15	0.3	2.2–2.7
P (mmol/L)	1.57	0.16	0.96–2.1
PTH (pmol/L)	3.61	2.24	1.3–6.8
Kidney function			
Cr (μmol/L)	26	6.21	21–65
Bun (mmol/L)	3.9	1.29	1.5–7
Thyroid function			
T3 (nmol/L)	2	0.31	1.1–3.9
T4 (nmol/L)	100	24	45.3–223.9
TSH (μIU/L)	3	1.8	0.64–6.27

Sera from patients with β-thalassemia major (β-TM) were subjected to various biomarker evaluations in the medical diagnostic lab at Shenzhen Children’s Hospital (SZCH). The serum biomarker concentrations of patients with β-TM were compared to the corresponding normal ranges according to the laboratory standards at SZCH

**Fig. 2:**
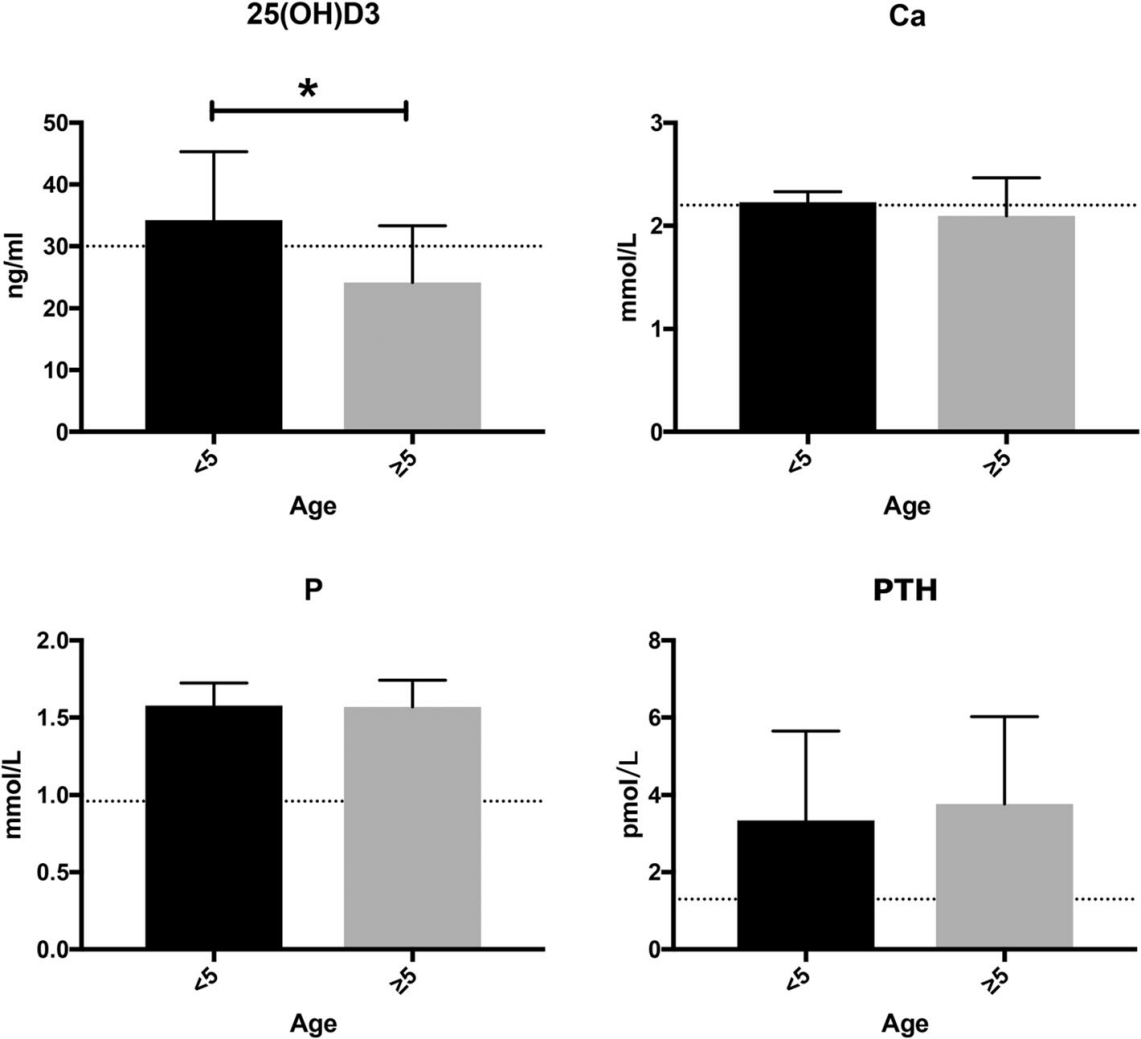
Evaluation of bone metabolism in patients with β-thalassemia major. Serum biomarkers of bone metabolism, including 25-hydroxy vitamin D3 (25(OH)D3), calcium (Ca), phosphorus (P), and parathyroid hormone (PTH), were compared between patients ≥5 and < 5 years old (**p < 0.01, 34.25 ± 11.06 vs. 23.05 ± 9.95 ng/ml, Mann-Whitney *U* test)

Next, we evaluated these biomarker concentrations with respect to age. In analyses of bone metabolism, the mean serum concentration of 25(OH)D_3_ was significantly higher in patients < 5 yo, compared to those aged ≥5 yo (**p < 0.01, 34.25 ± 11.06 vs. 23.05 ± 9.95 ng/ml, Mann-Whitney *U* test). Moreover, the mean serum concentration of 25(OH)D_3_ remained within the normal range among younger patients but was below the normal range in older patients. No significant age-related differences were observed in other markers of bone metabolism (e.g., serum calcium, phosphate, and PTH) (Fig. 2).

**Fig. 3:**
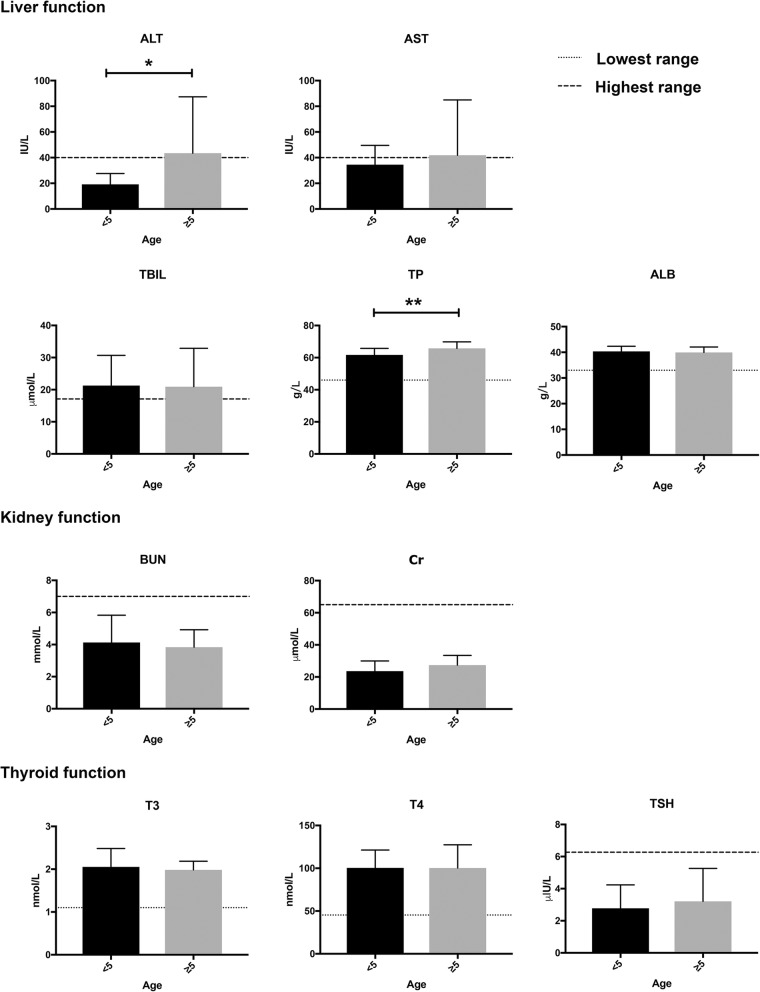
Laboratory evaluations the liver, kidney, and thyroid functions in patients with β-thalassemia major. Serum biomarkers of liver, kidney, and thyroid functions were assessed in patients ≥5 and < 5 years of age (**p < 0.01, 61.73 ± 4.06 vs. 65.77 ± 4.08 g/L, Mann-Whitney *U* test)

All patients were found to be hepatitis B and C seronegative. Accordingly, the serum levels of ALT, AST, TBIL, TP, and ALB were screened as markers of liver function. Patients < 5 yo had a significantly lower mean ALT concentration, compared to older patients (*p < 0.05, 19.17 ± 2.44 vs. 43.45 ± 9.82 IU/ml, Mann-Whitney *U* test). A significant increase of TP level was observed in the older patient group (**p < 0.01, 61.73 ± 4.06 vs. 65.77 ± 4.08 g/L, Mann-Whitney *U* test). However, no significant age-related differences were observed in the serum levels of the other liver function markers. Similarly, the serum concentrations of Cr and Bun and of T3, T4, and TSH were measured as markers of kidney and thyroid functions. Again, no significant age-related differences were observed (Fig. 3).

## Discussion

Although optimized blood transfusion and chelation protocols have led to significant improvements in survival among patients with β-TM over the last few decades, the complications associated with long-term blood transfusion remain a major factor affecting the quality of life in this population. Notably, osteoporosis is one of the most common complications observed in patients with β-TM [12], and previous studies of patients with thalassemia have described decreases in vitamin D and calcium levels and consequent reductions in bone intensity and defects in bone metabolism [13, 14]. However, the interaction between the vitamin D and calcium statuses and the associated risk of bone disease development in β-TM patients remain uncertain.

Vitamin D is a very important factor in both calcium and bone metabolism and, together with calcium, plays essential roles in bone development and bone maintenance. Previous studies found that thalassemia patients who had received multiple blood transfusions exhibited significant reductions in vitamin D levels of approximately 90%. In these patients, the upregulated absorption of iron leads to a significant reduction in the absorption of calcium [14–18]. Still, many factors other than vitamin D deficiency can cause hypocalcemia in patients with thalassemia; these include hypoparathyroidism, decreased vitamin D and calcium intakes, impaired vitamin D or calcium absorption, and iron overload [19–21]. However, vitamin D deficiency itself remains the leading cause of bone diseases in β-TM patients and may be exacerbated by reduced participation in outdoor activities due to anemia and skeletal malfunction [12, 22].

Both the severity of vitamin D deficiency and risk of severe bone diseases are associated with age. This causation remains unclear. However, previous study suggested this might be associated with more indoor activities or a decreased of overall vitamin D intake in older patients [6]. Although vitamin D and/or calcium supplementation are recommended to prevent osteopenia and severe bone diseases [12, 14, 16, 23], the optimal timing of these interventions has not been studied. Our findings were consistent with those of previous studies wherein older patients with thalassemia exhibited more severe vitamin D deficiency and thus faced a greater risk of developing osteopenia and other skeletal diseases (e.g., bone fractures). Furthermore, thalassemia patients younger than 5 yo in our study maintained relatively normal vitamin D levels, consistent with previous studies of complications due to thalassemia. We further note that as vitamin D and PTH maintain a reciprocal relationship, an elevated serum PTH level may imply a deficiency in vitamin D production. However, our study found only a slight and non-significant increase in the serum PTH concentrations of patients ≥5 yo, compared to those < 5 yo.

The development of endocrinopathies in patients with thalassemia and the factors influencing disease progression and severity remain under investigation [24]. Consistent with many previous reports, our study findings reinforce the existence of a positive correlation between age and serum ferritin concentrations in thalassemia patients. Although this latter parameter is included in decisions regarding chelation therapy, it may also be influenced by other factors such as inflammation, liver damage, and vitamin C deficiency. Moreover, adolescents with higher levels of ferritin face a higher risk of endocrine disorders such as hypogonadism, diabetes mellitus, hypoparathyroidism, and lifelong short stature [25–27]. In this study, we revealed a positive association between age and the serum ALT level, a marker of liver function, in our patient sample. Various biomarkers of liver function, including the serum bilirubin, ALT, AST, and ALB concentrations, correlate with the findings of T2* magnetic resonance imaging (MRI), a technique used to evaluate the severity of hepatic iron overload. However, China has not developed clear guidelines for the timing of T2* MRI in children with thalassemia. The clinical findings from our study suggest that iron overload may become apparent beyond 5 years of age, suggesting that T2* MRI of the liver may be considered at this time.

Regular blood transfusion and chelation therapy can greatly reduce complications due to iron overload, and therefore iron chelators such as DFO are prescribed to reduce iron accumulation in patients with thalassemia. However, these agents may be associated with hypocalcemia [4]. Nearly 60% of patients in our study were receiving regular iron chelation therapy with multiple chelating agents. Seven of the 12 patients who never received chelation therapy were ≥ 5 yo, despite previous findings that the delayed initiation of chelation therapy is associated with a higher serum ferritin level and, consequently, more frequent endocrine complications [28–31]. Moreover, patients with high blood concentrations of ALB may fail to respond to calcium or vitamin D supplementation therapy [27]. In our study, we observed a significant increase in the serum TP level in children ≥5 yo, compared to those < 5 yo. However, the small sample size of this study may have precluded the determination of a significant age-related difference in ALB levels.

Hypoparathyroidism due to iron overload is commonly observed in patients with thalassemia. This endocrine disorder has also been identified as a main cause of hypocalcemia. Interestingly, previous reports indicated reduced PTH levels in patients with thalassemia major and suggested that these patients would benefit from vitamin D and calcium supplementation [3, 19–21, 32, 33]. Taken together, the findings from our and previous studies suggest that early and effective treatment should be administered to improve bone health.

Our study had some limitations of note, including a relatively small sample size which may have precluded our ability to reach a statistically significant threshold. We did not consider the effects of some possible covariates that may have influenced the levels of vitamin D and other biomarkers, such as the nutritional status and physical activity. Moreover, we did not compare the results obtained in children with β-TM to those of healthy children. Finally, we were unable to exclude any possible disturbances that might affect vitamin D and calcium metabolism. Longer-term studies involving more patients should be conducted to validate the present results.

## Conclusion

In conclusion, our data reinforce previously published reports of vitamin D deficiency as a common manifestation in patients with thalassemia major, and particularly the strong association of this deficiency with age. However, the optimal timing of intervention remains uncertain. Importantly, our findings will guide clinicians in the appropriate timing of interventions to prevent severe complications of β-TM in pediatric patients.

## Data Availability

Not applicable.

## Abbreviations

25(OH)D_3_: 25-hydroxy vitamin D3
ALB: albumin
ALT: alanine aminotransferase
AST: aspartate aminotransferase
BUN: blood urea nitrogen
Cr: creatinine
DFO: desferrioxamine
MRI: magnetic resonance imaging
PTH: parathyroid hormone
T3: triiodothyronine
T4: thyroxine
TBIL: total bilirubin
TM: Thalassemia
TP: total protein
TSH: thyroid-stimulating hormone
yo: year old
